# Demographic characteristics of patients using a fully integrated psychosocial support service for cancer patients

**DOI:** 10.1186/1756-0500-2-253

**Published:** 2009-12-15

**Authors:** Donald M Sharp, Mary B Walker, Julie S Bateman, Fiona Braid, Claire Hebblewhite, Teresa Hope, Michael Lines, Andrew A Walker, Leslie G Walker

**Affiliations:** 1 The Institute of Rehabilitation, Hull York Medical School, University of Hull, 215 Anlaby Rd, Kingston upon Hull, East Yorkshire, HU3 2PG, UK; 2 The Oncology Health Centre, Queen's Centre for Oncology & Haematology, Hull and East Yorkshire Hospitals NHS Trust, Castle Hill Hospital, Castle Road, Cottingham Hull, HU16 5JQ, UK

## Abstract

**Background:**

Psychosocial support services are an important component of modern cancer treatment. A major challenge for all psychosocial services is the achievement of equity of use. Previous studies in the UK have found that women of higher socio-economic status with breast cancer were over-represented amongst those accessing support services. People with other cancer diagnoses, those from socio-economically deprived areas, and men, were under-represented.

**Findings:**

The Oncology Health Service, Kingston Upon Hull, UK, delivers fully integrated psychosocial support and interventions. To assess equity of access in this service, a cross-sectional study of all patients with cancer accessing the service during a 5 day period was carried out. One hundred and forty-five patients attended. Forty four percent were male, and the types of cancer were broadly in the proportions expected on the basis of population prevalence (breast cancer 22%, colorectal cancer 21%, lung cancer 16%). Sixty six percent came from the three most deprived quintiles of the Townsend deprivation Index.

**Conclusions:**

The fully integrated Oncology Health Service in Hull is accessed by a more diverse range of patients than previously reported for other services, and is an example of a model of service by which socially equitable use of psychosocial support in the National Health Service might be achieved.

## Background

Despite major advances in the treatment of cancers, and improvements in supportive and palliative care, the diagnosis and treatment of cancer continue to cause wide-ranging distress to patients and their families. High levels of psychosocial and psychiatric morbidity continue to be widely reported in the United Kingdom in patients with cancer, as well as in their families [1,2]. In addition to impoverishing quality of life, treatment outcome and prognosis may be associated with clinically significant psychological distress [3-5].

There are notable socio-economic inequalities in cancer care in the UK; in screening uptake, stage at first presentation, response to treatment and use of psychosocial support services. This is a challenge to cancer care services regarding the effectiveness of psychosocial support services provided for people with cancer and their families.

A range of psychosocial support services have been developed in the UK; from services independent of mainstream NHS oncology services, through partially integrated services, to psychosocial support services which are fully integrated with mainstream oncology provision. However, a previous review [6] found that only 23-29% of patients accessing support services were male. Others have noted that psychosocial support services are accessed predominantly by white, middle class women with breast cancer [7-9].

Consequently cancer patients who are, male, diagnosed with other cancer types, and from areas of social deprivation are not accessing and receiving potentially beneficial psychosocial support. Since many of these findings of inequity of use were published, psychosocial support services for cancer patients have developed in the UK. However, none of the more recently developed services have to date reported on equity of use.

In 1999, a unique Oncology Health Service was established in Kingston upon Hull. Full descriptions of the Service, together with the rationale, have been published [10,11]. The Oncology Health Service is staffed by a specially trained and supervised multi disciplinary team comprising Clinical and Research Nurse Specialists (Behavioural Oncology), and Clinical Psychologists, and is fully integrated physically, functionally, financially and managerially with other parts of local NHS oncology provision. The Oncology Health Service is integrated within the Cancer Centre in Kingston Upon Hull which provides all oncological diagnostic, treatment and follow up services for the full range of cancer patients. Patients and their families can access the Oncology Health Service either by traditional referral or via an open access drop-in centre which is staffed by the Specialist Behavioural Oncology Nurses. Patients can access the drop-in centre without appointment or prior arrangement as they wish. The Oncology Health Service has two principal clinical aims. Firstly, by providing readily available psychosocial support and by nature of the fully integrated service, the opportunity to resolve patients' concerns quickly and efficiently, much of the clinically significant distress noted above can be prevented [10,11]. Secondly any clinically significant distress which does arise can be treated efficiently without the requirement to refer patients to tertiary services outside of the Cancer Centre. Given the problems with equity of access previously noted for psychosocial support services, it is important to evaluate whether the fully integrated Oncology Health Service in Hull produces a more equitable pattern of use by people with cancer and their families. The aim of this research, therefore, was to obtain up-to-date information about the characteristics of service users in terms of factors germane to equity of use namely, gender, cancer type and indices of social deprivation.

## Materials and methods

Approval to carry out the audit was obtained from Hull and East Yorkshire Hospitals NHS Trust. All participants gave written informed consent.

### Study design

This was a cross sectional study of all patients with cancer attending the service during a consecutive 5-day period in June 2006 (telephone contacts with patients were not included). As routine activity monitoring indicated no seasonal variation in patient attendance, and to ensure minimal effect on the clinical operation of the Service the 5 day period (one working week) was chosen. Although many relatives also access the service and receive support and psychological interventions, they were not included in this study.

### Materials

A research proforma was constructed, to obtain reliable information about sociodemographics, clinical history and patient satisfaction. The proforma was completed in collaboration with the patient and with reference to case notes where appropriate. The methodology therefore comprised patient survey and medical and administrative record review. Social deprivation was assessed using the Townsend Deprivation Index, a measure of socio-economic deprivation [12] which was derived from post-code using 2001 Census data.

## Results

Percentages throughout are reported as valid percent and have been rounded to the nearest whole number.

### Participants

Excluding relatives, 145 patients attended the Centres during the 5-day period of the study and 135 (93%) of these gave written consent to participate in the audit. Two patients (1%) were too ill to participate, one (1%) had learning difficulties and could not give valid consent, and four (3%) were unable to participate because of other commitments. Three (2%) patients declined to participate.

### Gender

Fifty-six percent of patients accessing the service were female and 44% were male.

### Socio-demographics

The mean age of the patients was 61 years (median 62, range 31-90). Seventy-four percent were married or cohabiting, and 14% were widowed.

Sixty-two percent had not had any tertiary education. Twenty-two percent had a university degree. Thirty percent were retired, 33% had a manual occupation, 14% had a clerical/administrative post, and 17% had a professional occupation.

### Social Deprivation

Expressed as quintiles the Townsend Deprivation Index showed that 66% of the patients came from the three most deprived quintiles, 22% from the most deprived quintile.

### Clinical History

Twenty-two percent had breast cancer, 21% had colorectal cancer, 16% had lung cancer, and 8% had prostate cancer. The remaining 33% had a range of cancer diagnoses. Patients with the full range of cancer diagnoses attended.

Seventy-seven percent of patients were suffering from primary cancer. Fifty-one percent had undergone surgery for the current episode; 72% had received, or were receiving, chemotherapy; 38% had received, or were receiving, radiotherapy and 21% were receiving hormone therapy.

### Type of Attendance

The majority of patients, 66%, attended as "drop-ins" (that is, they did not have, nor require, an appointment), 10% were seen as inpatients in one of the wards, and the remainder attended by appointment. In 13% of cases, this contact was their first contact. The median number of previous visits was 8; the mode was 3 and the range was 1 to over 100 attendances.

### Patient satisfaction with the Oncology Health Service

One-hundred percent of the patients were satisfied with the service they had received from the Oncology Health Service (95% were "very satisfied" and 5% were "satisfied"). Furthermore, 92% of patients were satisfied with their overall treatment in the cancer services (68% were "very satisfied" and 24% were "satisfied").

## Discussion

National guidance has emphasised the need for cancer services to promote equity of use [13]. This investigation has demonstrated a high level of equity of use in terms of sociodemographics and clinical characteristics. Previous reviews of other support services have found that only 23-29% of patients accessing support services are male [6,9], whereas in this audit 44% were male. Furthermore, previous reports have found that psychosocial support services were accessed preferentially by patients with breast cancer [7-9], whereas only 22% of the patients accessing the Oncology Health Service had breast cancer and the full range of other cancer diagnoses were represented in the proportions expected. A previous review of a cancer counselling service [7] reported that the largest proportion of people accessing the service were from social classes A, B and C1, this representing a significant class bias towards affluence. In this audit a measure of social deprivation, a more direct and pertinent measure of poverty than social class, was derived from postcode according to national census data. The Townsend scores for the Oncology Health Service showed that 66% of the patients came from the three most deprived quintiles, 22% from the most deprived quintile.

Generally, this audit has shown that the Oncology Health Service is used by men and women, young and old, from all socioeconomic backgrounds, and with all types of cancer. The findings were found for a psychosocial support service which is fully integrated physically, functionally, managerially and financially with mainstream oncology provision, and provides for open unrestricted access via a drop in centre. Indeed, the largest proportion of patients attending the Oncology Health Service did so as "drop in" patients.

Furthermore, all 135 respondents were "satisfied" or "very satisfied" with their treatment in the Oncology Health Centres, and a significant proportion (92%), were "satisfied" or "very satisfied" with their overall treatment in the cancer services, attesting to the previously indentified relationship between attention to psychosocial needs and patient satisfaction [14]. There are limitations to the current study, however, in that, in keeping with previous reports on equity of use of psychosocial services [6,7] a simple audit design was used. More robust conclusions may be supported in future research which employs direct randomised comparisons of differing models of psychosocial service delivery.

## Conclusion

This audit has demonstrated that a more socially and clinically diverse patient population use the fully integrated Oncology Health Service than those attending other services previously reported in the literature. Given the notable use of the drop in service which is central to the Oncology Health Service model, it is possible that unrestricted drop in access of this type may eliminate some of the barriers to accessing psychosocial support services previously reported.

## Competing interests

The authors declare that they have no competing interests.

## Authors' contributions

LGW and DMS were responsible for the conception, design and conduct of the study. They conducted the analysis of the data and the interpretation of that analysis. They drafted the manuscript and contributed to it's critical review and revision. MBW, AAW, JSB, FB, CH, TH & ML were involved in the design of the study, the acquisition of data and the interpretation of the analysis of the data. All also contributed to the critical review and revision of the manuscript.

All Authors read and approved the final manuscript.
